# KeyRecs: A keystroke dynamics and typing pattern recognition dataset

**DOI:** 10.1016/j.dib.2023.109509

**Published:** 2023-08-21

**Authors:** Tiago Dias, João Vitorino, Eva Maia, Orlando Sousa, Isabel Praça

**Affiliations:** Research Group on Intelligent Engineering and Computing for Advanced Innovation and Development (GECAD), School of Engineering, Polytechnic of Porto (ISEP/IPP), 4249-015 Porto, Portugal

**Keywords:** Typing behavior, Biometric authentication, Anomaly detection, Machine learning

## Abstract

Keystroke dynamics can valuably contribute to the development of intelligent authentication systems by enabling a single and continuous authentication process in a passive and non-intrusive manner by continuously verifying a user's identity. This work describes the KeyRecs dataset, which contains fixed-text and free-text samples of user typing behavior and demographic information of the participants age, gender, handedness, and nationality. The keystroke data was obtained from 99 participants of various nationalities who completed password retype and transcription exercises. The recorded samples consist of inter-key latencies computed in a digraph fashion measuring the time between each key press and release during an exercise. KeyRecs can be leveraged to improve the recognition of authorized users and prevent unauthorized access in biometric authentication software.

Specifications Table

**Table:** 

Subject	Artificial Intelligence
Specific subject area	Keystroke dynamics, typing pattern recognition, anomaly detection, and classification with machine learning models
Type of data	Table
How the data were acquired	The data acquisition process began by setting up a job in Prolific, which is an online platform that connects researchers with study participants for academic research studies. After the participants consented to participate in the study, they were given access to a website that followed a MEAN stack, which included MongoDB for storage, Express for routing, Angular for user interface and Node.js as runtime environment. The website contained a demographic data form and multiple typing exercises, collecting the time between each key press and release from each participant's own keyboard input.
Data format	Filtered Analyzed
Description of data collection	The data collection process included password retype and transcription exercises to gather fixed-text and free-text samples, respectively. The time between each key press and release during an exercise was processed to provide features with inter-key latencies. Regarding the demographic data, the participants were asked to provide their age, gender, handedness, and nationality.
Data source location	The source of the typing patterns was each participant's own keyboard input. The online participants were from various locations: Poland, Portugal, Greece, Italy, United Kingdom, Hungary, South Africa, Czech Republic, Estonia, Spain, France, Myanmar, Slovenia, Indonesia, Pakistan, Germany, Israel, Latvia, Mexico, and United States of America
Data accessibility	Repository name: Zenodo Data identification number (DOI): 10.5281/zenodo.7886742 Direct URL to data: https://doi.org/10.5281/zenodo.7886742[1]

## 1 Value of the Data

• This dataset provides recordings with the inter-key latencies of user typing behavior and keystroke dynamics in password retype and transcription exercises, alongside demographic information.
• Researchers, Machine Learning (ML) engineers, and software developers can benefit from this dataset to train, validate, and test ML models for robust typing pattern recognition solutions and biometric authentication software.
• The fixed-text and free-text samples can be used for anomaly detection tasks to recognize authorized users and prevent illegal logins.
• The recorded typing patterns follow a digraph fashion and can be repurposed for the clustering and classification of users that present a similar behavior.
• This dataset can be combined with additional data recordings to create more complete and extensive datasets and improve the generalization of ML models.

## 2 Objective

In the cybersecurity field, authentication remains a significant area of research. In recent years, Multi-Factor Authentication (MFA) has become increasingly popular for offering a resilient multi-layer access security protocol [2]. Keystroke dynamics has emerged as a reliable biometric method that uniquely identifies each individual, making it a suitable security protocol for MFA [3].

In the keystroke authentication space, recent works include the usage of keystroke dynamics samples to create biometric-based authentication systems utilizing Machine Learning and Deep Learning algorithms [4], [5], [6]. The authors of such works [4], [5], [6] achieve promising results, making this a relevant field to further explore. Their prediction models utilize datasets such as Clarkson II [6], and Keystroke Dynamics Dataset presented by Buffalo University [7] and Carnegie-Mellon University [4]. These datasets mainly include keystroke fixed-text and free-text samples from a diverse set of participants and take into consideration recordings from different keyboards. However, their scope focuses on keystroke information, disregarding human characteristics that may have impact on keystroke patterns. In addition, keystroke dynamics datasets are quite scarce, hence the need to increase the amount of variable data reliably collected for this purpose [3].

This work introduces KeyRecs dataset, which is composed of keystroke information and demographic attributes of multiple participants, collected via two different typing exercises. The exercises involved password retyping and text transcription, leading to fixed-text and free-text samples that can be used for resilient single and continuous authentication. KeyRecs dataset was created to contribute to the development of robust typing pattern recognition software and biometric authentication systems. By providing comprehensive data that truly depicts user typing behavior, ML models can be trained to recognize authorized users. This is especially important given the increasing prevalence of cyberattacks, which often rely on password cracking and unauthorized access [8]. This dataset serves as a valuable resource for researchers and developers across different fields including biometric authentication, security, and behavioral research. It provides an opportunity to explore and analyze typing behavior and keystroke dynamics-based authentication, which can be used to create more effective security systems better equipped to thwart cyber-attacks.

## 3 Data Description

The proposed dataset contains the inter-key latencies computed during the password retype and transcription exercises, as well as complementary demographic information of the participants, divided into three comma-separated values (CSV) files: *fixed-text.csv, free-text.csv*, and *demographics.csv*. The following subsections detail the contents of each file.

### 3.1 Fixed-text File

The *fixed-text.csv* file contains the data samples of all the inter-key latencies of each participant in the password retype exercise, structured into 19773 rows and 50 columns. Table 1 provides a description of each feature. Since the characters that the participants had to type in each repetition were fixed, columns 4 to 49 are in a sequential order and in the same format.

**Table 1:** Column descriptions of the fixed-text file.

#	Name	Description
1	participant	The identifier of the participant
2	session	The number of the session
3	repetition	The number of times the participant has repeated the typing in the current session
4-49	(D|U)(D|U).key1.key2	The inter-key latency of the current repetition, in seconds, between the pressing down (D) or releasing (U) of a key (key2), relative to the previous one (key1)
50	total.time	The total time of the current repetition, in seconds

### 3.2 Free-text File

The *free-text.csv* file contains the data samples of all the inter-key latencies of each participant in the transcription exercise, structured into 562584 rows and 9 columns. Table 2 provides a description of the features represented by each column. Since the characters that the participants had to type were not fixed, the rows are organized in a sequential order, specifying the pairs of character keys utilized by each participant at a time.

**Table 2:** Column descriptions of the free-text file.

#	Name	Description
1	participant	The identifier of the participant
2	session	The number of the session
3	key1	The first character key of the current pair
4	key2	The second character key of the current pair
5-9	(D|U)(D|U).key1.key2	The inter-key latency, in seconds, between the pressing down (D) or releasing (U) of a key2 relative to key1

### 3.3 Demographics File

The *demographics.csv* file contains the demographic attributes provided by each participant. Table 3 provides a description of the features represented by each column. This information is complementary to the data of the fixed-text and free-text files.

**Table 3:** Column descriptions of the demographics file.

#	Name	Description
1	participant	The identifier of the participant
2	handedness	The hand preference of the participant
3	age	The age of the participant
4	gender	The gender of the participant
5	nationality	The country of origin of the participant

## 4 Experimental Design, Materials and Methods

The proposed dataset comprises data collected from 99 participants recruited in Prolific [9], an online platform that facilitates study participation. In addition to the recorded keystroke dynamics of the participants during password retype and transcription exercises, their demographic information was also requested, which includes age, gender, handedness, and nationality. The following subsections describe the experimental design, execution, and setup of the study and provides a data quantification analysis of the dataset.

### 4.1 Design and Execution

**Fig. 1:**
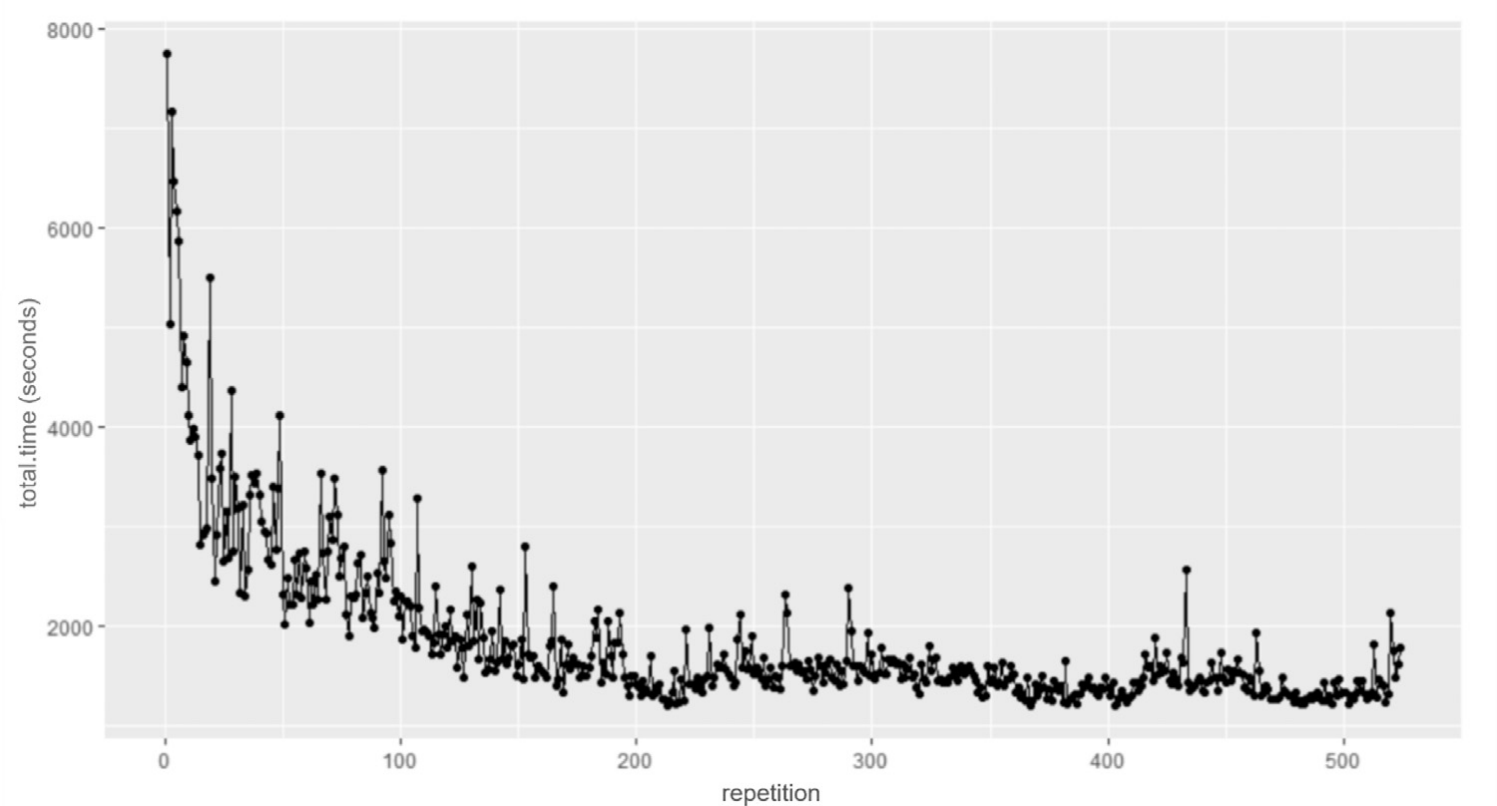
Password typing repetition estimation.

The first exercise of the study consisted of writing the password *vpwjkeurkb*, until becoming familiarized with it to produce stable and realistic keystroke data. However, as the password for the exercise was created at random and then utilized by all participants, it is safe to assume none of them were previously familiarized with it. Prior to asking the participants to perform the exercise, the authors decided to conduct an experiment to estimate the number of times that a password should be written until reaching a performance plateau. In Fig. 1, it is depicted the result of this experiment with the total time per typing repetition in milliseconds. The results suggest that at the 100-repetition mark, a performance plateau starts to form, and at the 200-repetition mark, there is no significant performance improvement. As such, the 200-repetition measurement was utilized as reference for the number of times each participant would have to type the password previously mentioned, to ensure that the typing performance of the participant became gradually more realistic. For this reason, the exercise was divided into two typing sessions with 100 repetitions each, to gather samples before and after the participants get familiarized with the password.

The second exercise consisted of transcribing 10 simple literature passages collected from public websites. Typically, this exercise is conducted using a questionnaire and monitoring the user's typing behavior. However, since there is less control over the number of keystrokes produced by each participant when answering open-ended questions, and considering the intrusiveness associated with the use of a keylogger to monitor each participant's activity, the authors relied on text transcription to collect the free-text data. Findings on the differences between transcribed and freely composed text support the design of this exercise by suggesting that transcription keystroke features are 2–3 ms slower than freely written text, encouraging researchers to use transcription exercises to gather this kind of data [10]. This exercise was divided into two similar typing sessions, where the participants transcribed 5 literature passages in each.

Afterwards, the designed and implemented study was executed by 100 participants gathered on Prolific. After collecting the data, the authors assessed the raw typing information to find possible mistakes. The validation consisted in ensuring that in the fixed-text exercise, the participants had typed the wanted characters and that in the free-text exercise, no invalid or unknown characters had been written. In this filtering stage, since one of the participants did not pass the verifications, those data samples had to be excluded, leaving the dataset with only data collected from 99 participants. Afterwards, a feature computation stage was performed to compute the inter-key latencies of each exercise of each remaining participant [11].

**Fig. 2:**
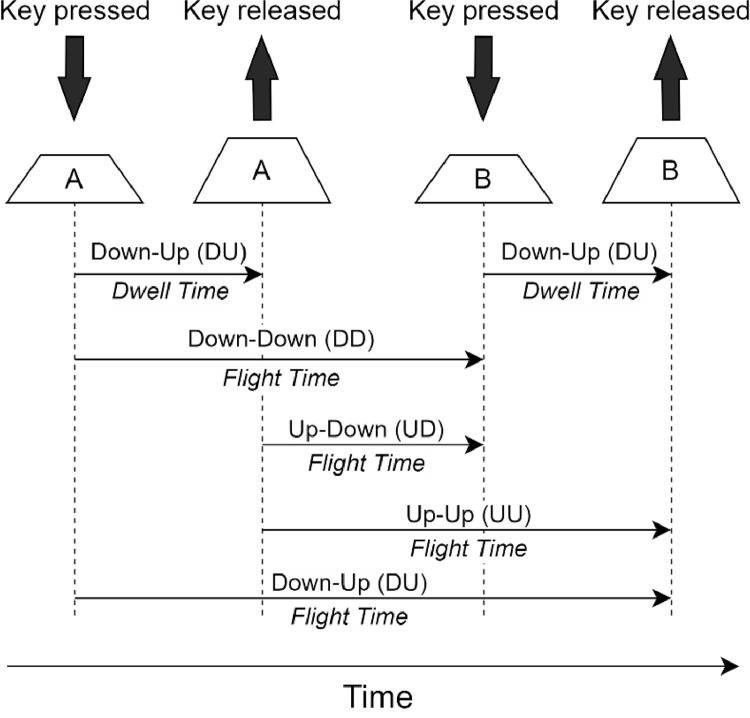
Inter-key latencies computed in a digraph fashion.

The inter-key latencies represent the number of seconds between the possible combinations of two events: a key is pressed down (D), and a key is released (U). These were computed in a digraph fashion, by accounting for different time instances, as demonstrated in Fig. 2. For instance, for the characters “A” and “B”, this will result in several features: “DU.A.A”, “DU.B.B”, “DD.A.B”, “UD.A.B”, “UU.A.B”, “DU.A.B”.

### 4.2 Setup

The authors decided that the conducted study would be realized online with each participant using their own equipment for the experiment. Since the authors did not find any existing survey tool capable of retrieving keystroke data, a website was developed and deployed to provide the exercises and record the data in a reliable manner. The website follows a MEAN technology stack: MongoDB for persistent storage, Express for the data access layer, Angular for the presentation layer, and Node.js as runtime environment. The applications that make up the website were hosted in the cloud, resorting to Docker Swarm to achieve load balancing through a NGINX reverse proxy and facilitating containerization.

### 4.3 Data Quantification

The dataset under consideration comprises of roughly 1.6 million keystrokes gathered from the 99 participants. On average, each participant generated around 15,994 key events, with 4611 on the fixed-text and 11,383 on the free-text task. The time taken by the participants to complete both exercises varied significantly, with the majority taking between 14 to 56 min. To maintain impartiality, ensure participant motivation, and data quality, identical conditions regarding incentives were provided to all participants during the study, and none were offered incentives to achieve any particular outcome.

**Fig. 3:**
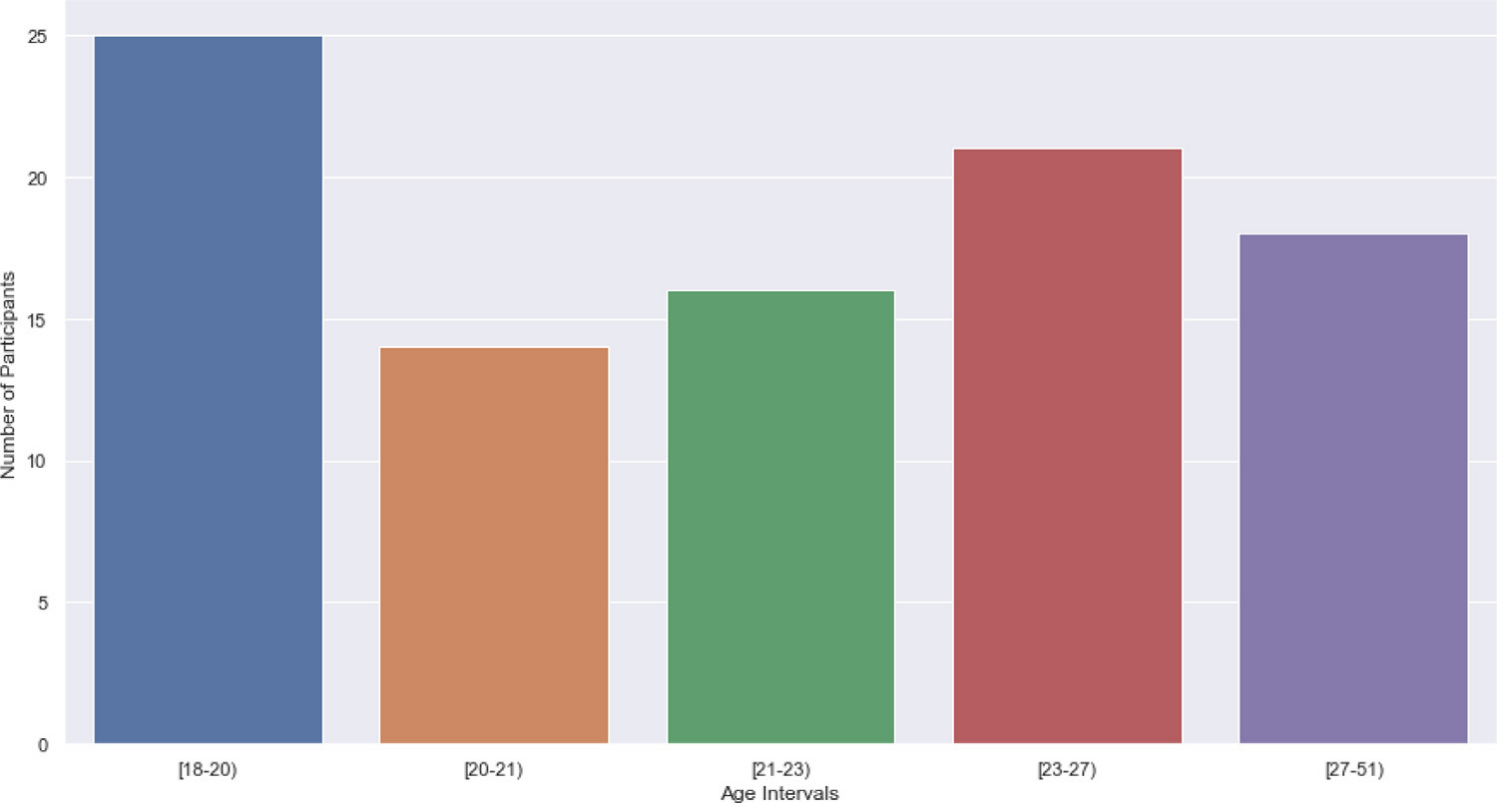
Ages of the participants.

Regarding the demographic information, from the included participants, 39 were female and the remaining were male, and 8 were left-handed and the remaining right-handed. The study was performed mostly by individuals between the age of 18–20, as depicted in Fig. 3. Nonetheless, there is still a significant quantity of older participants.

**Fig. 4:**
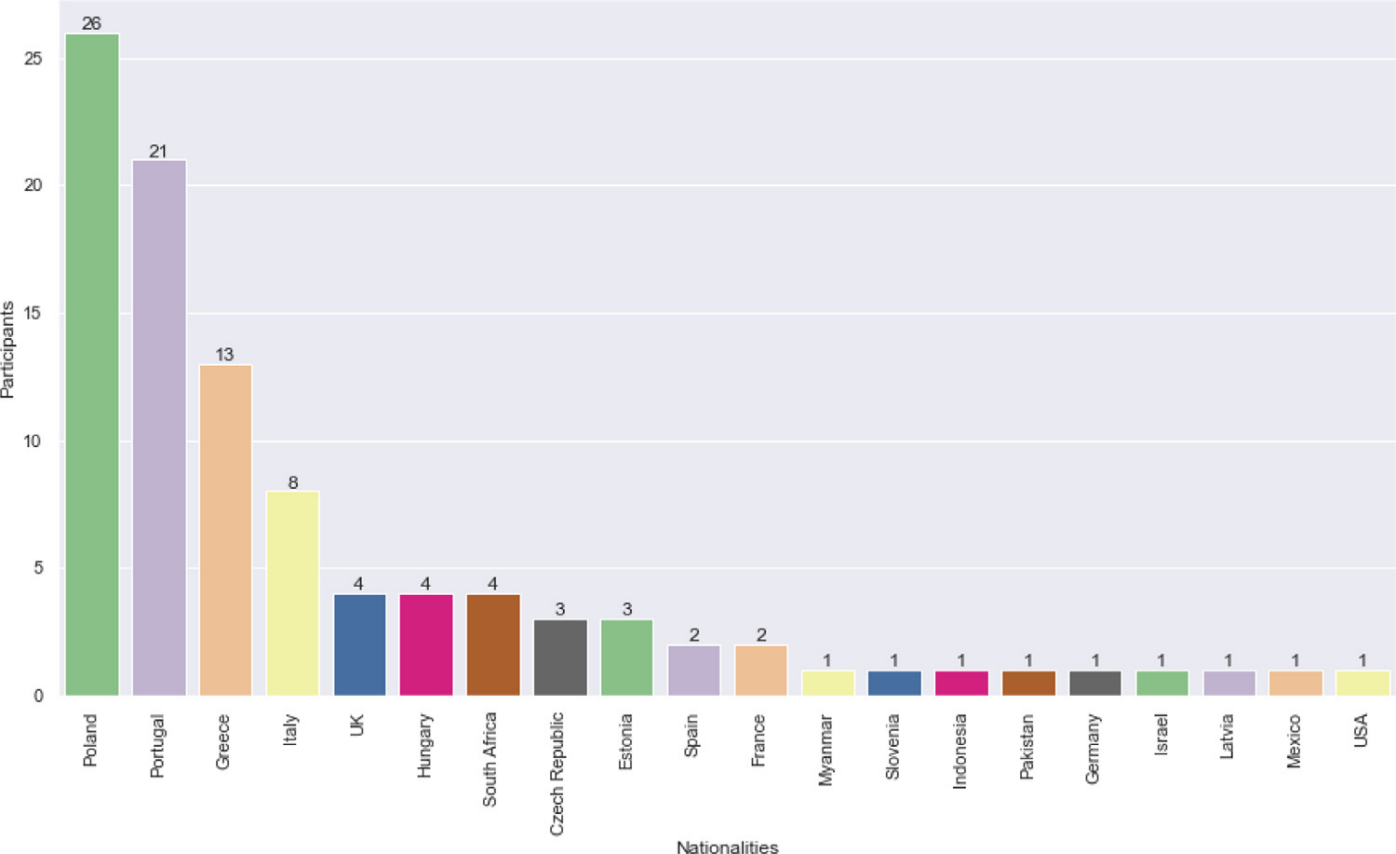
Nationalities of the participants.

As shown in Fig. 4, the study included keystroke data from participants of 20 different nationalities, with most being from Poland. This diverse representation adds to the richness of the dataset across multiple geographical regions, as keyboard layouts vary by location. Moreover, cultural differences between participants from different regions may contribute to their unique typing behaviors. By incorporating a range of nationalities, the analysis offers valuable insights into the influence of demographics and cultural diversity on typing behavior.

## Ethics Statements

This work did not directly involve data from any animal experiments, nor social media platforms. An informed consent was obtained from the online participants for the collection of their typing patterns and demographic attributes for the creation of this publicly available dataset.

## CRediT authorship contribution statement

**Tiago Dias:** Conceptualization, Methodology, Software, Investigation, Data curation, Formal analysis. **João Vitorino:** Methodology, Software, Investigation. **Eva Maia:** Validation, Supervision, Investigation. **Orlando Sousa:** Validation, Resources, Investigation. **Isabel Praça:** Conceptualization, Resources, Project administration, Funding acquisition.

## Data Availability

KeyRecs Keystroke Dynamics Dataset (Original data) (Zenodo).

## References

[1] Dias T., Vitorino J., Maia E., Praça I. (2023).

[2] Ometov A., Bezzateev S., Mäkitalo N., Andreev S., Mikkonen T., Koucheryavy Y. (2018). Multi-factor authentication: a survey. Cryptography.

[3] Raul N., Shankarmani R., Joshi P. (2020).

[4] Chang H.-C., Li J., Wu C.-S., Stamp M. (2021).

[5] Chang H.-C., Li J., Stamp M. (2021).

[6] Li J., Chang H.-C., Stamp M. (2021).

[7] Sun Y., Ceker H., Upadhyaya S. (2016). 2016 IEEE International Workshop on Information Forensics and Security (WIFS).

[8] Yamauchi T., Akao Y., Yoshitani R., Nakamura Y., Hashimoto M. (2018). 2018 IEEE Conference on Dependable and Secure Computing (DSC).

[9] “Prolific Quickly find research participants you can trust.” https://www.prolific.co/ (Accessed 8 March 2023).

[10] Killourhy K.S., Maxion R.A. (2012). Proceedings of the 2012 Workshop on Learning from Authoritative Security Experiment Results.

[11] Yaacob M.N., Idrus S.Z.S., Ali W.N.A.W., Mustafa W.A., Jamlos M.A., Wahab M.H.A. (2020). A review on feature extraction in keystroke dynamics. J. Phys. Conf. Ser..

